# A phenome-wide bidirectional Mendelian randomization analysis of atrial fibrillation

**DOI:** 10.1093/ije/dyac041

**Published:** 2022-03-15

**Authors:** Qin Wang, Tom G Richardson, Eleanor Sanderson, Matthew J Tudball, Mika Ala-Korpela, George Davey Smith, Michael V Holmes

**Affiliations:** Clinical Trial Service Unit & Epidemiological Studies Unit, Nuffield Department of Population Health, University of Oxford, Oxford, UK; Medical Research Council Integrative Epidemiology Unit, University of Bristol, Bristol, UK; Computational Medicine, Faculty of Medicine, University of Oulu and Biocenter Oulu, Oulu, Finland; Center for Life Course Health Research, University of Oulu, Oulu, Finland; Medical Research Council Integrative Epidemiology Unit, University of Bristol, Bristol, UK; Population Health Sciences, Bristol Medical School, University of Bristol, Bristol, UK; Novo Nordisk Research Centre Oxford, Oxford, UK; Medical Research Council Integrative Epidemiology Unit, University of Bristol, Bristol, UK; Population Health Sciences, Bristol Medical School, University of Bristol, Bristol, UK; Medical Research Council Integrative Epidemiology Unit, University of Bristol, Bristol, UK; Population Health Sciences, Bristol Medical School, University of Bristol, Bristol, UK; Computational Medicine, Faculty of Medicine, University of Oulu and Biocenter Oulu, Oulu, Finland; Center for Life Course Health Research, University of Oulu, Oulu, Finland; NMR Metabolomics Laboratory, School of Pharmacy, University of Eastern Finland, Kuopio, Finland; Medical Research Council Integrative Epidemiology Unit, University of Bristol, Bristol, UK; Population Health Sciences, Bristol Medical School, University of Bristol, Bristol, UK; Clinical Trial Service Unit & Epidemiological Studies Unit, Nuffield Department of Population Health, University of Oxford, Oxford, UK; Medical Research Council Integrative Epidemiology Unit, University of Bristol, Bristol, UK; Medical Research Council Population Health Research Unit at the University of Oxford, Oxford, UK; National Institute for Health Research, Oxford Biomedical Research Centre, Oxford University Hospital, Oxford, UK

**Keywords:** Atrial fibrillation, Mendelian randomization, stroke, proteins

## Abstract

**Background:**

The prevalence of atrial fibrillation (AF) is increasing with an aging worldwide population, yet a comprehensive understanding of its causes and consequences remains limited. We aim to assess the causes and consequences of AF via a bidirectional Mendelian randomization (MR) analysis.

**Methods:**

We used publicly available genome-wide association study (GWAS) summary data, centralized and harmonized by an open GWAS database. We assessed the genetically predicted effects of 5048 exposures on risk of AF, and the genetically predicted effects of genetic liability to AF, on 10 308 outcomes via two-sample MR analysis. Multivariable MR analysis was further conducted to explore the comparative roles of identified risk factors.

**Results:**

MR analysis suggested that 55 out of 5048 exposure traits, including four proteins, play a causal role in AF (*P* <1e-5 allowing for multiple comparisons). Multivariable analysis suggested that higher body mass index, height and systolic blood pressure as well as genetic liability to coronary artery diseases independently cause AF. Three out of the four proteins (DUSP13, TNFSF12 and IL6R) had a drug prioritizing score for atrial fibrillation of 0.26, 0.38 and 0.88, respectively (values closer to 1 indicating stronger evidence of the protein as a potential drug target). Genetic liability to AF was linked to a higher risk of cardio-embolic ischaemic stroke.

**Conclusions:**

Our results suggest body mass index, height, systolic blood pressure and genetic liability to coronary artery disease are independent causal risk factors for AF. Several proteins, including DUSP13, IL-6R and TNFSF12, may have therapeutic potential for AF.

Key Messages

- The prevalence of atrial fibrillation (AF) is increasing with an aging worldwide population, yet a comprehensive understanding of its causes and consequences remains limited. In this work, we leveraged >10 000 publicly available genome-wide association study summary data and undertook a phenome-wide bidirectional Mendelian randomization (MR) analysis to comprehensively examine the causes and consequences of AF.
- Stringent pipelines were specifically built for protein and non-protein exposures to identify the causal risk factors for AF, and multivariable MR analyses were further employed to assess the comparative causal effects of risk factors.
- MR analysis provides evidence supporting the independent effects of adiposity, height, systolic blood pressure and liability to coronary artery disease in the aetiology of AF. Several proteins, including DUSP13, IL-6R and TNFSF12, may represent therapeutic potential for preventing AF.
- In the reverse direction, MR provided evidence that genetic liability to AF may increase the risk of cardio-embolic ischaemic stroke but not of other subtypes of ischaemic stroke, e.g. large-artery atherosclerosis and small-vessel disease.

## Introduction

Atrial fibrillation (AF) is the most common cardiac rhythm disorder, affecting 1–2% of the population in Europe and North America.^1^^,^^2^ The prevalence and incidence of AF are expected to increase further due to the aging population, and it has been predicted that Europeans aged >40 yrs have a one in four lifetime risk of developing AF.^1^ AF is associated with an increased risk of stroke, myocardial infarction, heart failure, and mortality, posing considerable challenges to public health and the economy.^1^^,^^3^^,^^4^

Despite remarkable advances in anti-arrhythmic drugs, ablation procedures and stroke-prevention strategies, AF remains an important cause of death and disability in middle-aged and elderly individuals.^5^ Clinical management of patients with AF is currently guided by stroke risk parameters, AF pattern and symptoms.^5^ However, more than half of patients with AF remain symptomatic despite adequate anticoagulation and rate control.^5^ Better understanding of the mechanisms leading to AF and the interplay of AF and its associated complications is warranted.

Observational studies have reported numerous risk factors to associate with AF risk, including obesity, smoking, alcohol consumption, diabetes, hypertension, reduced lung function, coronary artery disease and heart failure.^1^^,^^4^ Mendelian randomization (MR) analyses^6^ have suggested multiple causal risk factors for AF, including thyroid dysfunction,^7^ adiposity,^8^^,^^9^ higher birthweight,^10^ raised blood pressure,^11^ being taller^12^ and lower circulating soluble IL-6 receptors.^13^ On the other hand, studies assessing the consequences of genetic liability to AF are limited and a recent MR study reported a lack of a causal role of AF genetic liability on risk of Alzheimer’s disease.^14^ Despite these and other efforts geared at identifying individual risk factors for AF, studies applying a hypothesis-free approach to systematically assess the causes and consequences of AF have yet to be conducted. Here, we leveraged thousands of publicly available genome-wide association study (GWAS) summary data and undertook a phenome-wide bidirectional MR analysis to comprehensively examine the causes and consequences of AF, which might provide an important basis to guide future strategies in preventing and treating AF and avoiding AF-related sequelae.

## Methods

### Data sources

We used publicly available GWAS summary data, which are curated and centralized by the Medical Research Council Integrative Epidemiology Unit (MRC-IEU) open GWAS database [https://gwas.mrcieu.ac.uk], and can be accessed via R package ‘TwoSampleMR’.^15^^,^^16^ GWAS summary data for atrial fibrillation reported for European descents, consisting of 60 620 cases and 970 216 controls, were used in the present study.^17^

### Traits filtering

**Figure 1:**
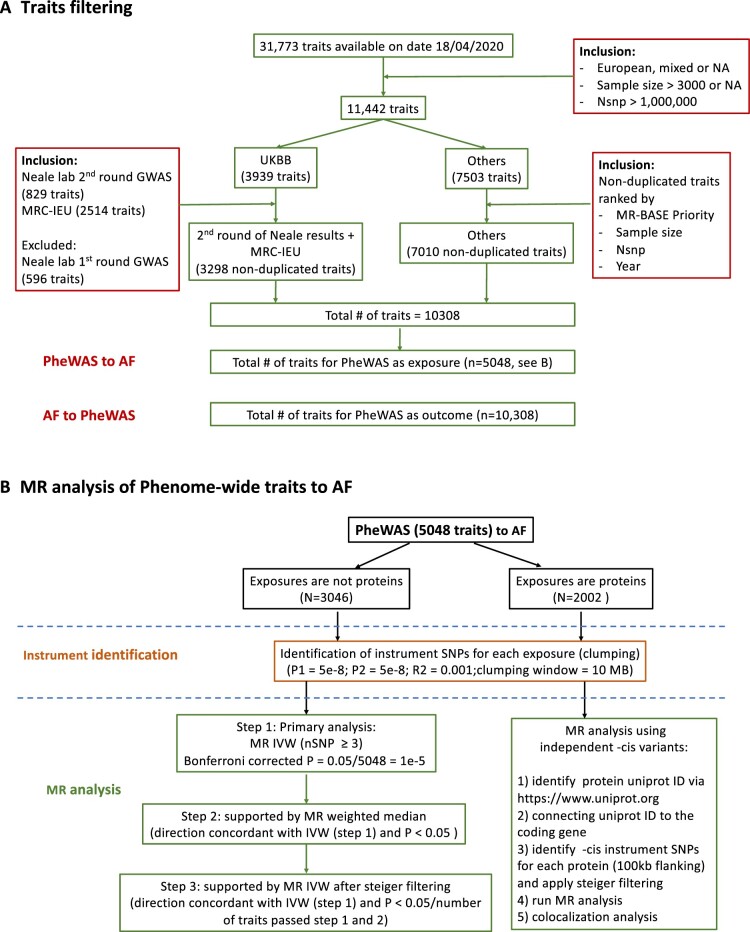
Analysis flow. A) traits filtering; B) analysis flow in conducting Mendelian randomization (MR) analysis of phenome-wide traits to atrial fibrillation. AF, atrial fibrillation; GWAS, genome-wide association study; IVW, inverse-variance weighted; MRC-IEU, Medical Research Council Integrative Epidemiology Unit; P1, significance threshold for index SNP; P2, significance threshold for secondary SNPs; PheWAS, phenome-wide association study; SNP, single-nucleotide polymorphism

There were 31 773 traits with GWAS summary data accessible via ‘TwoSampleMR’ package on 18 April 2020. Traits pre-filtering was applied (Figure 1A) as follows: studies were included if they were primarily based on European descendants, had sample sizes over 3000 [to include as many traits as possible, e.g. to incorporate a proteomics GWAS (≈3000 proteins)^18^ with a sample size of 3301, and also have adequate sample sizes to generate reliable instruments] and had over 1 million genetic variants (to maximize the availability of the genetic instruments). In total, GWAS summary data for 3298 traits analysed using UK Biobank data released by Neale’s lab (second round) and MRC-IEU, and 7010 traits analysed by other consortiums or studies, are included here. Of note, the second round GWAS release from Neale’s lab was used as the sample sizes are in general larger than the first round and also have a better curated analysis pipeline [http://www.nealelab.is/uk-biobank/ukbround2announcement]. This filtering resulted in 5048 exposure and 10 308 outcome traits for the MR analyses (Figure 1A).

### MR analysis from phenome-wide traits (5048 exposures) to AF

The MR analysis flow of phenome-wide exposures to atrial fibrillation is shown in Figure 1B.

#### Genetic instruments for exposures

Clumping was applied to establish independent genetic variants for each individual exposure. Clumps are formed around central ‘index variants’ which must have p-value no larger than 5e-8. Index variants were chosen greedily, starting with the lowest p-value. Secondary hits were identified if they were within the clumping window (10 Mb) of an index single-nucleotide polymorphism (SNP), reached GWAS significance (p <5e-8) and had a low linkage disequilibrium (LD) with the index SNP (r^2^ <0.001 based on 1000 Genomes phase 3 data from European descendants).

As binary traits from UK Biobank data were analysed in linear regression models, associations of the genetic variants with binary traits were scaled to log odds by multiplying a scaling factor 1/(μ * (1 − μ)), where μ = n_case_/(n_case_ + n_conrol_).^19^ Whenever applicable, genetic associations with quantitative traits were reported in standard deviation (SD) and binary traits in log odds.

#### MR analysis

After genetic instruments were identified for each exposure, the associations of these genetic variants with atrial fibrillation were extracted. If the genetic variants were not directly available in the outcome GWAS, proxies with r^2^ > 0.8 were used based on 1000 Genomes phase 3 data from European descendants and, in the case of no proxies being identified (e.g. due to imputation of different platforms), the variants were then removed. In total, there were 5048 exposure-outcome pairs with instrument variants available in both exposure and outcome GWAS. Two-sample MR analyses were performed via five different methods, including inverse-variance weighted (IVW), weighted median, MR Egger, simple mode and weighted mode.^21^ To ensure the robustness of the findings, we also assessed the F statistic for each of the exposures to avoid bias arising from weak genetic instruments. In general, genetic risk scores including multiple variants spanning the genome are preferred as the instrument for complex traits (e.g. non-protein measures), and on the other hand *cis* variants located around the protein coding gene are typically considered as being more reliable instruments for proteins.^20^ Here, different MR analysis pipelines (Figure 1B) were used for protein versus non-protein exposures to apply context-specific analytical approaches.

When exposures were complex traits (e.g. non-proteins), we selected all SNPs across the genome which associated with the trait at GWAS significance. MR estimates from the IVW method were treated as the primary results. The estimates were considered robust if they were supported by a three-stage approach: step 1—there were more than three genetic variants for use in the instrument (minimal number of variants required to perform all five MR methods). Given the number of tests conducted, we used *P* < 0.05/5048 (number of total exposures) = 1e-5 as a heuristic to guide findings that were further explored in steps 2 and 3; step 2—primary IVW estimates were directionally concordant with those of weighted median, and *P* (weighted median) <0.05; step 3—primary IVW estimates were directionally concordant with IVW estimates after steiger filtering, and *P* (steiger) <0.05/number of traits passing steps 1 and 2. Steiger filtering was applied to ensure that each instrument variant explained larger variances of exposures than outcomes, thus increasing the reliability of the assumed direction of causality.^16^ For those exposure-outcome pairs that passed the three-stage sensitivity test, we further examined the consistency of the five MR methods, as different MR methods have different assumptions and limitations, and thus if all methods were consistent, we would have better confidence to infer causality and argue against pleiotropy (or other forms of bias).^21^

When exposures were proteins, we aimed to identify -*cis* variants to proxy the proteins.^22^ We manually matched each protein to an unique Uniprot ID [https://www.uniprot.org]. Then, we connected Uniprot ID to coding genes. For each protein, -*cis* instrument variants were identified if they were located within the coding gene (100 kb flanking), were associated with the protein at *P* <5e-8 and explained more variance in the proteins than the outcome trait. Here, we used Wald ratio or IVW estimates as the primary MR approach. Similarly, Bonferroni corrected *P *< 0.05/5048 = 1 x 10^-5^ was used to guide interpretation of the findings. For each protein that was suggested to have a causal role for AF in the MR analysis, we further conducted colocalization analysis between the protein GWAS and AF GWAS at the protein coding gene (100 kb flanking of the leading *cis*-pQTL) and used posterior probabilities of sharing one common causal variant (H4) to guide interpretation of colocalization.^23^

### MR analysis from AF to phenome-wide traits (10 308 outcomes)

#### Genetic instruments for exposure (atrial fibrillation)

Using a similar approach as above, we identified 111 independent SNPs (between SNP LD r^2^ < 0.001; association with AF *P* < 5 x 10^-8^) as the genetic instruments for AF.

#### MR analysis

We extracted the associations of these 111 SNPs with each individual outcome trait. In total, there were 10 308 exposure-outcome pairs with instrument variants available in both exposure and outcome GWAS. Similar to the above section described for non-protein exposures, we used IVW estimates as the primary results and results were considered robust only when they fulfilled the three-stage analysis approach described above. For the positive findings, we also performed weighted median, MR Egger, simple mode, weighted mode and an additional method for coarsened exposures.^21^^,^^38^ The coarsening-adjusted method facilitates us to interpret the MR estimates in a liability scale (e.g. interpreted in terms of standard deviation changes in AF liability) whereas the unadjusted estimates are on the log-odds scale.^38^

### Multivariable analyses

As many risk factors are typically correlated with each other, multivariable MR (MVMR) was used to explore the genetically predicted independent effects of these risk factors with regard to AF. We first grouped risk factors by whether univariable MR provided evidence supporting a relationship with AF into different categories, based on whether they were sharing the same feature [e.g. body mass index (BMI) and height were grouped in the category of anthropometry, and systolic and diastolic pressures (SBP, DBP) were grouped in the category of blood pressure]. MVMR analyses were then conducted for traits within each individual category to elucidate their comparative genetically predicted effects on the risk of AF. The independent risk factors within each individual category were then selected and combined in a final MVMR model. We estimated conditional F statistics for the exposures in the MVMR models using the method described by Sanderson *et al.*,^24^ and also provided the corresponding F statistics in the univariable MR (UVMR) models for a comparison.^25^

## Results

### MR analysis from phenome-wide traits to atrial fibrillation

**Figure 2:**
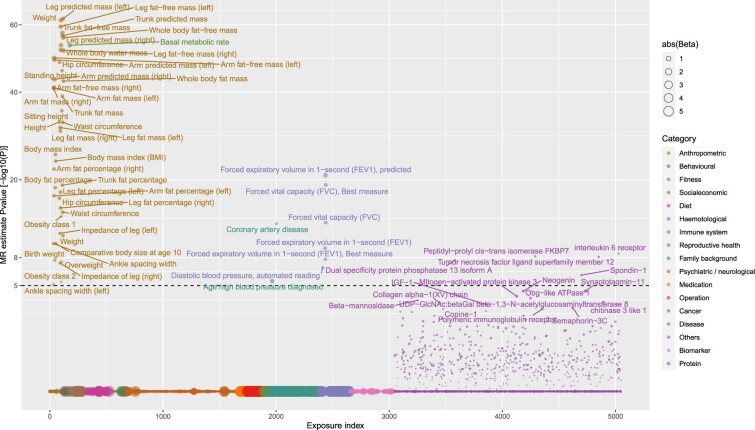
Genetically predicted effects of phenome-wide traits and risk of atrial fibrillation. The Y-axis denotes the common logarithm (log10) of the *P*-values of the Mendelian randomization (MR) estimates, and the X axis denotes the number or index of the exposure traits. Symbols are coloured according the category of exposure traits, and the symbol sizes are proportional to the absolute values of the MR estimates (based on inverse-variance weighted method). The horizontal black line corresponds to the Bonferroni corrected *P* = 0.05/5048 traits = 1e-5. For display purposes, the associations that were not supported by any of the three-stage tests (see Figure 1B), we set the *P*-values equal to one (indicating absence of reliable evidence).

**Figure 3:**
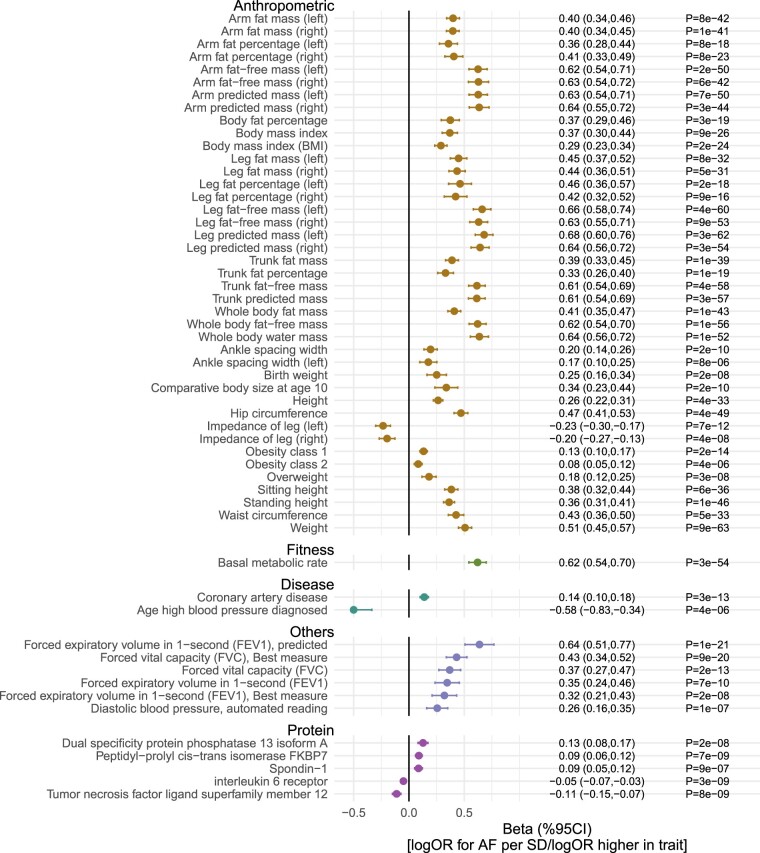
Genetically predicted effects of selected traits and risk of atrial fibrillation (AF). Mendelian randomization effect estimates represent differences in natural logarithm of odds ratio (logOR) for AF per unit higher in exposure trait. Traits presented are those that surpass multiple testing (Bonferroni *P* <0.05/5048 exposures = 1e-5) in Figure 2. CI, confidence interval; SD, standard deviation

MR analyses suggested that out of 5048 exposures (3046 non-protein measures and 2002 proteins), 55 traits were putatively causal (Bonferroni *P* <1 x 10^-5^ from IVW MR) for the development of atrial fibrillation (Figures 2 and 3). The majority (50 of 55) were non-proteins, with most being related to anthropometry (e.g. height, fat mass, lean fat mass, waist, hip, height, birthweight, ankle spacing width and impedance of leg). In general, MR provided genetic support for relationships between these anthropometric traits and AF risk, except impedance of legs. Fat-free mass (in arms, legs, trunk and whole body) displayed an approximately 1.5 times larger magnitude [log odds ratio (OR) per SD higher anthropometric trait] of relative risk than corresponding fat mass measures. In addition to anthropometric traits, higher basal metabolic rate, genetic liability to coronary artery disease (CAD), respiratory traits [forced expiratory volume in 1 s (FEV1) and forced vital capacity] and higher diastolic blood pressure were also linked to higher AF risk. Moderate evidence of a potential causal effect was found for systolic blood pressure [log OR [95 CI%] per SD higher SBP = 0.25 (0.14, 0.36), *P* = 1 x 10^-5^]. In sensitivity analyses, the risk markers were consistently associated with AF across six different MR methods [five MR methods using all genetic variants and one using steiger filtered variants (IVW)] (Supplementary Figure S1, available as Supplementary data at *IJE* online**)**. The MR estimates for all 5048 exposures across the six methods are reported in Supplementary Table S1 (available as Supplementary data at *IJE* online). In addition, our results suggested that conventional cardiovascular risk factors, including apolipoprotein B, triglycerides, LDL cholesterol, glucose, HbA1C and type 2 diabetes, were not causal for AF (Supplementary Table S2, available as Supplementary data at *IJE* online).

**Figure 4:**
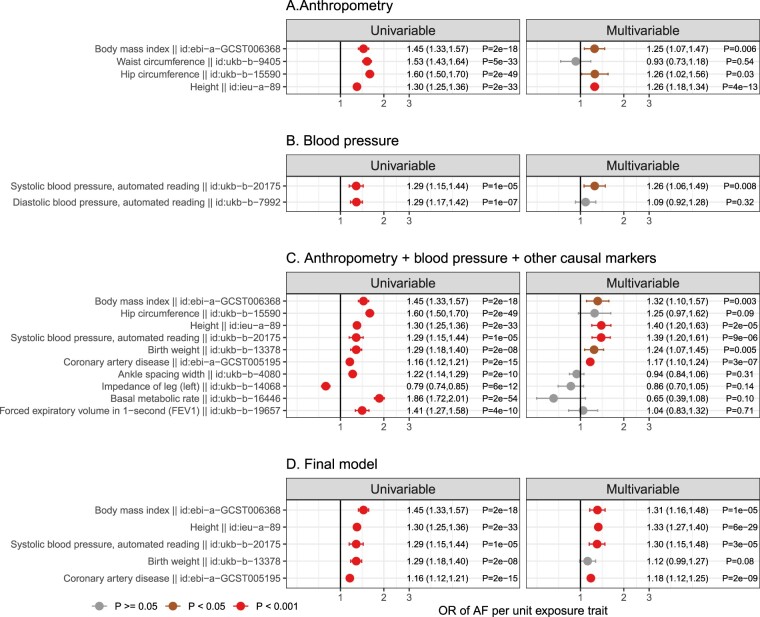
Genetically predicted independent effects of selected traits and risk of atrial fibrillation (AF). Step A, multivariable Mendelian randomization analysis (MVMR) of anthropometry traits. Effect estimates from univariable MR analysis (left) were compared with those from MVMR analysis (right). Step B, MVMR of blood pressure traits. Step C, the independent risk factors from step A (body mass index, hip and height) and B (systolic blood pressure) were selected and combined with additional risk factors in MVMR. Step D, independent risk factors from Step C were fitted in the final multivariable model. Associations with *P* ≥ 0.05 are coloured in grey, *P* <0.05 in brown and *P* <0.001 in red. OR, odds ratio

Multivariable MR was used to investigate the genetically predicted comparative effects of risk factors (Figure 4). We first fitted a multivariable model including markers showing evidence of causation with AF on univariable MR which were related to anthropometry, and the results implicated BMI, hip and height showing evidence of independent causal roles (Figure 4A). Similarly, we fitted a multivariable model for blood pressure traits and the results suggested that SBP was the underlying risk factor (Figure 4B). Finally, we selected the above risk markers (BMI, hip circumference, height and systolic blood pressure) and generated a multivariable model which also included other markers, including ankle spacing width, impedance of leg, basal metabolic rate, birthweight, CAD and forced expiratory volume (Figure 4C). The MVMR analysis provided evidence of genetically predicted independent effects of BMI, height, SBP and liability to CAD in the aetiology of AF (Figure 4C and D). The genetic correlations of these risk factors are reported in Supplementary Figure S2 (available as Supplementary data at *IJE* online), and the conditional F statistics of these exposures across the MVMR models and corresponding F statistics in the UVMR models are listed in Supplementary Table S3 (available as Supplementary data at *IJE* online).

**Figure 5:**
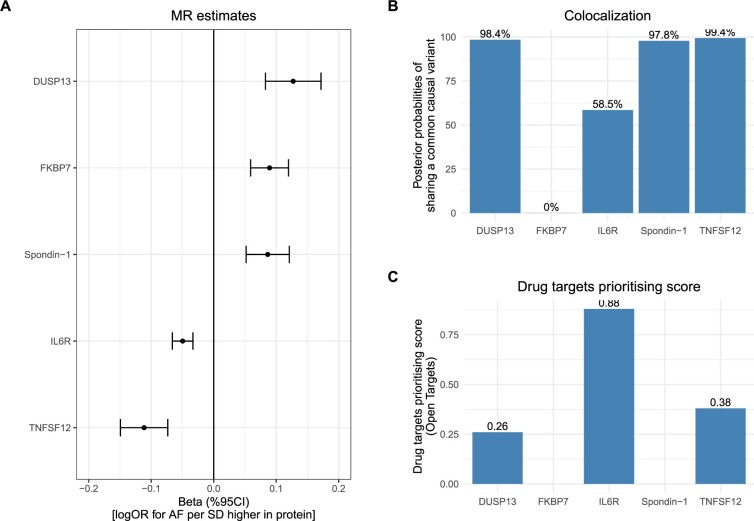
Genetically predicted effects of proteins and risk of atrial fibrillation (AF). A) Mendelian randomization (MR) estimates of proteins with risk of AF. B) Colocalization analysis demonstrating the posterior probability of circulating protein and AF sharing a common causal variant at the protein coding region. Additional details are provided in Supplementary Table S4 (available as Supplementary data at *IJE* online). C) Drug targets prioritizing score for the proteins. Data were obtained from Open Targets platform [https://www.opentargets.org]. For each protein, the top 40 associated traits or diseases are illustrated in Supplementary Figure S4, available as Supplementary data at *IJE* online. The platform allows prioritization of drug targets based on the strength of their association with a disease. It allows for the prioritization of targets by scoring target-disease associations based on evidence from 20 data sources. Similar data sources are grouped together into data types. The scores for the associations range from 0 to 1; the stronger the evidence for an association, the stronger the association score (closer to 1). A score of 0 corresponds to no evidence supporting an association. Missing data for two proteins in panel C corresponds to lack of available data. CI, confidence interval; DUSP13, dual specificity protein phosphatase 13 isoform A; FKBP7, peptidyl−prolyl cis−trans isomerase FKBP7; IL-6R, interleukin 6 receptor; logOR, natural logarithm of odds ratio; SPON1, spondin − 1; TNFSF12, tumour necrosis factor ligand superfamily member 12

In addition, among the 2002 proteins, 578 proteins had (steiger filtered) -*cis* instruments available and 423 of the 578 proteins had a single variant instrument. Here, MR analysis suggested that five proteins might have a causal role in AF, including higher levels of dual specificity protein phosphatase 13 isoform A (DUSP13), peptidyl−prolyl cis−trans isomerase FKBP7 (FKBP7) and spondin − 1 (SPON1), and lower levels of interleukin 6 receptor (IL-6R) and tumour necrosis factor ligand superfamily member 12 (TNFSF12) (Figure 5A). Colocalization analysis of circulating protein levels and risk of AF at the protein coding region further suggested that DUSP13, SPON1 and TNFSF12 had strong evidence of sharing a common causal variant [posterior probability (PP) of H4 ≥98%], and there was moderate evidence for IL-6R (PP of H4 = 59%) (Figure 5B;Supplementary Figure S3 and Supplementary Table S4, available as Supplementary data at *IJE* online). However, no colocalization evidence was observed for FKBP7 (PP of H4 = 0%). To characterize the therapeutic potential of modifying these proteins in preventing AF, we looked up the drug prioritizing scores in Open Targets platform [https://www.targetvalidation.org]. The results (Figure 5C;Supplementary Figure S4, available as Supplementary data at *IJE* online) suggested that three out of the four proteins (all other than spondin-1) had a moderate to strong drug prioritizing score for AF, ranging from 0.26 to 0.88. In particular, despite only moderate evidence of colocalization, IL-6R had the highest prioritizing score among these proteins of 0.88.

### MR analysis of genetic liability to atrial fibrillation on the human phenome

**Figure 6:**
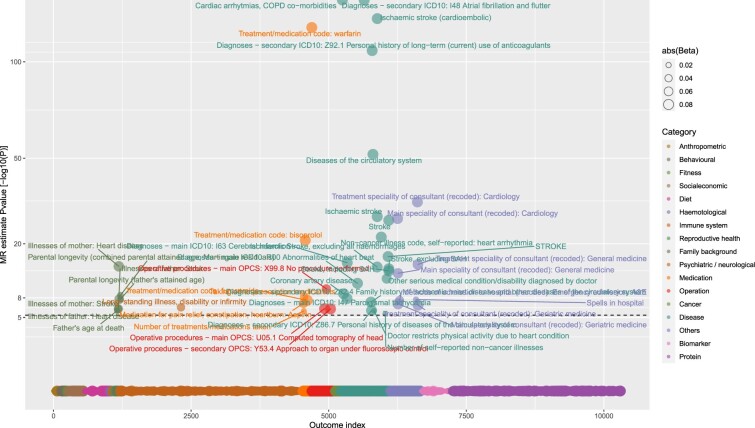
Genetically predicted effects of liability to atrial fibrillation (AF) and phenome-wide traits. The Y-axis denotes the common logarithm (log10) of the *P*-values of the Mendelian randomization (MR) estimates, and the X-axis denotes the number or index of the outcome traits. Symbols are coloured according the category of outcome traits and the symbol sizes are proportional to the absolute values of the MR estimates (based on inverse-variance weighted method). The horizontal black line corresponds to the Bonferroni corrected *P* = 0.05/10 308 traits = 5e-6. For display purposes, for the associations which were not supported by any of the three-stage sensitivity test, we set the *P-*values equal to one (indicating non-reliable). COPD, chronic obstructive pulmonary disease; ICD, International Classification of Diseases; OPCS, Office of Population Censuses and Surveys; SAH, subarachnoid hemorrhage

**Figure 7:**
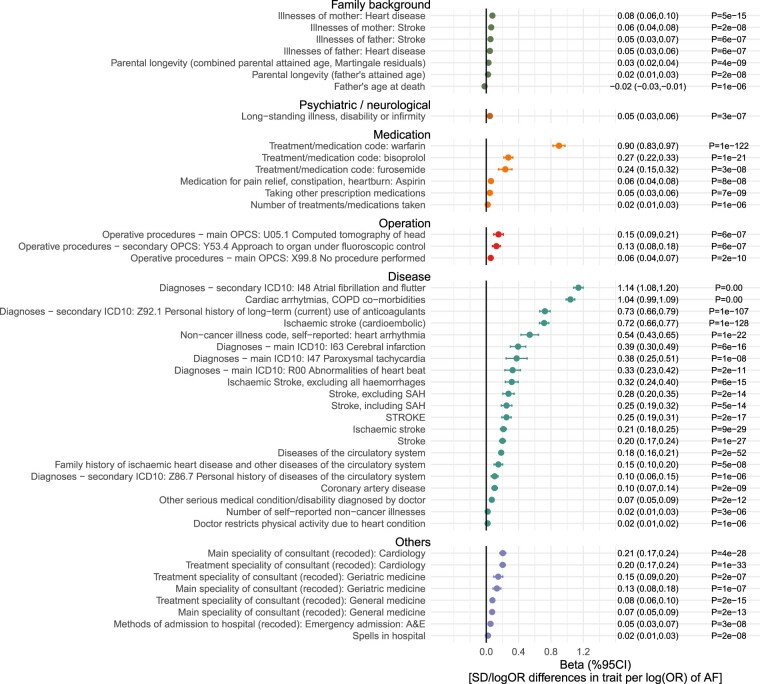
Genetically-predicted effects of liability to atrial fibrillation (AF) and selected traits. The estimates represent differences in traits per unit higher risk of atrial fibrillation. Traits presented are those that surpass multiple testing (Bonferroni *P* < 0.05/10 308 outcomes = 5e-6) in Figure 6. CI, confidence interval; COPD, Chronic obstructive pulmonary disease; ICD, International Classification of Diseases; OPCS, Office of Population Censuses and Surveys; OR, odds ratio; SD, standard deviation; SAH, subarachnoid haemorrhage

Out of 10 308 exposure-outcome pairs, MR analysis provided genetic evidence in support of 46 traits being the potential consequence of genetic liability to AF (*P* <5x10^–6^) (Figures 6 and 7). These traits include parental history of heart disease or stroke, medications in relation to anticoagulation (warfarin), heart rate and blood pressure control (bisoprolol and furosemide) and antiplatelet (aspirin), and also diseases related to coronary artery disease and stroke. Results were largely consistent across the six MR methods (Supplementary Figure S5, available as Supplementary data at *IJE* online). The MR estimates for all the 10 308 exposure-outcome pairs across the six different MR methods are reported in Supplementary Table S5 (available as Supplementary data at *IJE* online).

**Figure 8:**
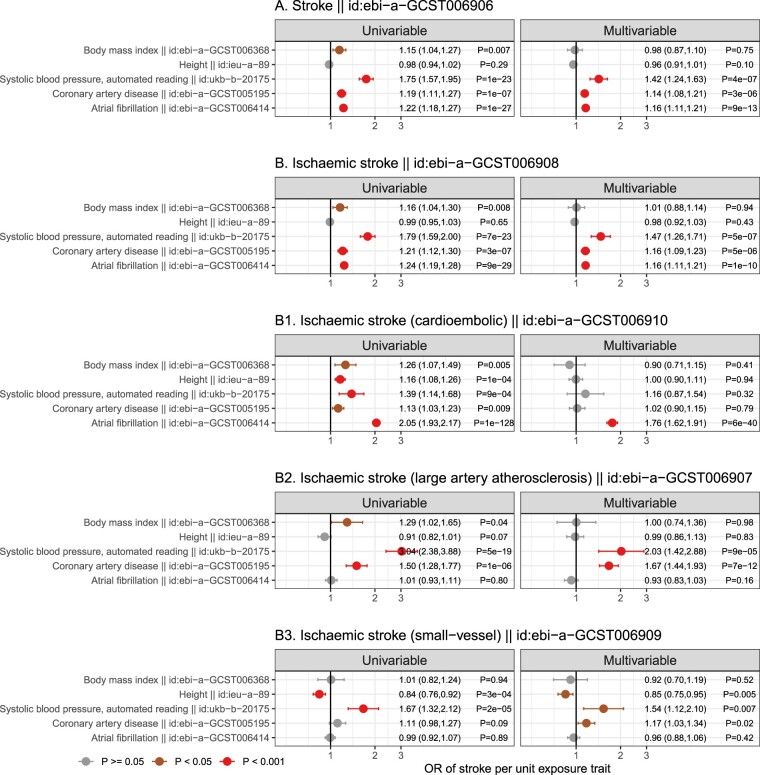
Genetically predicted independent effects of selected traits and risk of A) all stroke, B) ischaemic stroke and B1-B3) ischaemic stroke subtypes. Mendelian randomization (MR) estimates from univariable analyses (left) were compared with those from multivariable MR analyses (right). Associations with *P* ≥0.05 are coloured in grey, *P* <0.05 in brown and *P* <0.001 in red. OR, odds ratio

As the genetically predicted effects mostly related to stroke risk or medications (which is expected and serves as a positive control), we investigated whether liability to AF showed consistent association patterns across stroke types (Figure 8). In order to understand the degree to which genetic liability to AF contributes to stroke types and its mediating role, multivariable analyses incorporating BMI, height, SBP and CAD (risk factors showing evidence of causation for AF, Figure 4D) as the covariates were used. The conditional F statistics for each exposure across the models are shown in Supplementary Table S6 (available as Supplementary data at *IJE* online). For stroke overall (Figure 8A), the results supported that genetic liability to AF retained a potential causal effect in the multivariable MR model, and that the multivariable model further indicated that genetic liability to AF, SBP and liability to CAD each played a potentially independent causal role. A similar pattern (Figure 8B) was also seen for ischaemic stroke (the major type of stroke, accounting for around 85% of stroke cases globally).^26^ In exploring three subtypes of ischaemic stroke (comprising cardio-embolic ischaemic stroke, large-artery atherosclerotic stroke and small-vessel stroke), different patterns (Figure 8B1–B3) became evident. Genetic liability to AF displayed the largest magnitude of effect with risk of cardio-embolic stroke. In univariable analysis, all the risk factors displayed positive effects for cardio-embolic ischaemic stroke; however, in the multivariable model only genetic liability to AF retained an independent role [OR (95 CI%) = 1.96 ([1.81, 2.13), *P* = 3x10^–58^), suggesting AF might mediate the effects of the other risk factors on risk of cardio-embolic ischaemic stroke (Figure 8B1). For large-artery atherosclerotic stroke and small-vessel ischaemic stroke, null effects of genetic liability to AF were obtained in both univariable and multivariate models (Figure 8B2 and B3).

## Discussion

To systematically explore the causes and consequences of atrial fibrillation, we conducted a phenome-wide, bidirectional MR analysis of atrial fibrillation, spanning thousands of traits including anthropometric, behavioural and socioeconomic measures, diet, neurological factors, reproductive health, diseases, medication and operational codes, as well as a wide range of biomarkers and proteins. MR analyses provided evidence that adiposity (indexed by BMI), height, systolic blood pressure and coronary artery disease are direct causal risk factors for risk of AF, and that genetic liability to AF increases the risk of cardio-embolic ischaemic stroke, potentially mediating the effects of BMI, height, SBP and CAD. Several proteins, including circulating levels of IL-6 receptor, showed evidence of potential causation for AF, representing therapeutic potential.

In this work, multiple risk factors showed potential evidence of a role in the aetiology of AF and most of them were related to anthropometric measures. Our findings that measures of adiposity were potentially causally related to AF are in keeping with previous studies^8^^,^^9^ including those linking systolic blood pressure (a previously identified causal consequence of adiposity) to AF.^36^^,^^37^ In addition, our results implicate a detrimental effect of taller height on risk of AF (consistent with a prior study^12^). This suggests contrasting effects of height on different cardiovascular diseases. For example, prior MR studies have shown taller height to be protective of coronary artery disease,^27^^,^^28^ potentially mediated by a beneficial lipid profile, lower adiposity and better lung function.^12^^,^^27^^,^^28^ A detailed analysis of the relationship of height with stroke subtypes (Figure 8) revealed distinctive patterns with higher height increasing the risk of cardio-embolic stroke on univariable MR analysis, but lowering the risk of small-vessel ischaemic stroke. Multivariable MR analysis further suggests that the positive causal role of height for cardio-embolic stroke is likely to be mediated via its effect on increased risk of AF. Notably, these potentially divergent effects of height on risks of ischaemic stroke subtypes were obscured when using a composite endpoint (i.e. when using all stroke and ischaemic stroke), and underscores the importance of a detailed exploration of disease subtypes.

Among the approximately 2000 proteins investigated in this study, four were suggested to be causal for AF with three (DUSP13, TNFSF12 and IL6R) displaying medium to high drug prioritizing scores. Studies linking DUSP13 and TNSF12 to atrial fibrillation are limited; however, previous studies suggest that *DUSP13* gene expression was upregulated after stress stimulation in cardiomyocytes^29^ and that *TNFSF12* may be related to angiogenesis.^17^ Among these proteins, IL-6R showed the highest drug prioritizing score of 0.88. AF has been associated with various inflammation biomarkers, and with a previous study implicating NLRP3 inflammasome activation (which leads to 1 L-1β activation and consequently its downstream effects on IL6 acting through the IL6 receptor) in AF.^30^^,^^31^ Taken together our findings, which are consistent with a previous study,^13^ underlie the therapeutic potential of pathways downstream of IL-1β for treating AF. In line with the promising genetic findings, randomized conrolled trials (RCTs) have been conducted to assess the effects of IL-1β inhibitors in treating cardiovascular disease. In a recent phase III clinical trial, canakinumab, a monoclonal antibody inhibitor of interleukin-1 beta (IL1b) which has a license for rheumatological disorders, was shown to lower the risk of cardiovascular diseases.^32^ Also, a recent small pilot RCT (*N* = 24) of canakinumab in patients with persistent AF found a numerically lower incidence of AF recurrence at 6 months in the treatment arm as compared with placebo.^33^ These initial pilot data support potential future larger trials assessing the clinical feasibility of IL-1β inhibitors (and indeed IL6R inhibition) for treating AF.

Our MR analysis of genetic liability to AF on phenome-wide traits suggested that liability to AF leads to an increased risk of stroke and stroke-related medications. Our results of stroke subtypes provided evidence that genetic liability to AF is specifically contributing to cardio-embolic ischaemic stroke but not other ischaemic stroke subtypes, and it is this relationship that likely mediates the effects of body mass index, height, systolic blood pressure and liability to coronary artery disease on the risk of cardio-embolic ischaemic stroke. In contrast, a distinctive causal pattern was observed for the other two subtypes of ischaemic stroke, namely large-artery atherosclerosis and small-vessel disease, which are primarily dominated by blood pressure and the onset of coronary artery disease,^34^ independent of AF genetic liability.

The strength of this study lies in the hypothesis-free approach in assessing the bidirectional causal role of phenome-wide traits with AF, permitting the comprehensive evaluation and discoveries that we report. To ensure the robustness of the results, a three-stage sensitivity approach was designed for non-protein exposures, whereas only cis acting genetic variants were used to instrument protein exposures. To address multiple testing we used Bonferroni corrections, and the consistency of the findings was compared across six different MR methods. We acknowledge that sample overlap between exposure and outcome GWAS may induce overfitting in the case of weak instruments.^35^ However, this potential bias should be marginal given the adequate F statistics of the risk factors in the univariable MR model (Supplementary Tables S3 and S6). In addition, we used conditional F statistics to guide our MVMR analysis in minimizing bias from weak instruments. Almost all exposures had conditional F statistics ≥10 except the instrument for genetic liability to CAD which had a conditional F statistic of 9 (Supplementary Tables S3 and S6) and which demonstrated similar estimates on univariable and multivariable MR analyses, arguing against potential weak instrument bias. Our findings also facilitate further research to explore the possible non-linear causal relations between the reported risk factors and AF.

## Study limitation

Overall, despite over 5000 traits being used as exposures and over 10 000 traits being used as outcomes in this bidirectional MR analysis, we acknowledge that further positive findings may be revealed when more GWAS of detailed phenotypes and larger sample sizes become available. This could be the case for the negative findings here we observed for the conventional risk factors, e.g. lipids, HbA1c and type 2 diabetes. However, given the marginal beta effect estimates of these exposures (beta close to zero) and that *P* >0.05, it is possible that the observational associations of these risk factors with AF are confounded. Further, given the diverse association pattern between AF and stroke subtypes, further studies might investigate the causal role of AF with regard to haemorrhagic stroke (which may be mediated by anticoagulant therapies used in the treatment of AF). Of note, the MR estimates of AF on the risk of paternal diseases and longevity likely represent a shared genetic background between the study participants and their first-degree relatives, and thus should not be interpreted as evidence of causation. This is described in further detail elsewhere^39^ but in brief, we do not claim that genetic liability to AF directly alters risk of outcomes in first-degree relatives because genetically elevated liability to AF is only an approximation to unconfounded estimates within first-degree relatives. We also acknowledge that the current work is conducted using data from individuals of European ancestry and thus the results may not be generalized to other populations.

## Conclusions

Here we present a bidirectional MR study of atrial fibrillation, a common disease yet for which the pathophysiology remains poorly understood. Our comprehensive framework of assessment across thousands of phenotypes provided genetic evidence that adiposity, height, systolic blood pressure and liability to coronary artery disease represent potentially independent causal risk factors for AF. The replication of evidence supporting an effect of these established risk factors on AF reinforces the validity of our analytical framework. Besides these known factors, we also reported novel genetically predicted effects of multiple proteins (DUSP13, TNFSF12 and IL6R) on risk of AF, highlighting their therapeutic potential. In addition, we explored the effects of genetic liability to AF on a wide range of traits and diseases and our results suggest a heterogeneous pattern of effects with stroke subtypes, highlighting the need and value of detailed characterization of clinical outcomes.

## Ethics approval

Public summary data were used and therefore no ethics approval was required.

## Supplementary Material

dyac041_Supplementary_Data

## Data Availability

Data are publicly available at [https://gwas.mrcieu.ac.uk].
