# Supplementary material for: A phenome-wide bidirectional Mendelian randomization analysis of atrial fibrillation
Source: Int J Epidemiol. 2022 Mar 15;51(4):1153–66. doi: 10.1093/ije/dyac041 (PMC9365635; doi:10.1093/ije/dyac041)
Supplement: dyac041_Supplementary_Data [file dyac041_supplementary_data.zip › dyac041_Supplementary_Data/ije-2021-02-0287-File011.pdf]

Supplemental materials

Content

Supplementary tables

(All tables are presented in excel file)

Table S1. MR estimates from exposures to atrial fibrillation.
Table S2. MR estimates from typical cardiovascular risk factors to atrial fibrillation.
Table S3. F-stats for exposures in univariable and multivariable MR analysis to atrial fibrillation.
Table S4. Colocalization analysis for proteins and atrial fibrillation.
Table S5. MR estimates from atrial fibrillation to outcomes.
Table S6. F-stats for exposures in univariable and multivariable MR analysis to stroke and stroke subtypes.
Table S7. GWAS summary info for atrial fibrillation and significant exposure traits (P < 5e-6).
Table S8. GWAS summary info for significant outcome traits (P < 5e-6).

Supplementary figures

Figure S1. Sensitivity test for the significant findings in the MR analysis of phenome-wide traits to atrial fibrillation.
Figure S2. Genetic correlations of the risk factors.
Figure S3. Locuszoom plots for the proteins and atrial fibrillation.
Figure S4. Drug targets prioritising scores.
Figure S5. Sensitivity test for the significant findings in the MR analysis of atrial fibrillation to phenome-wide traits.

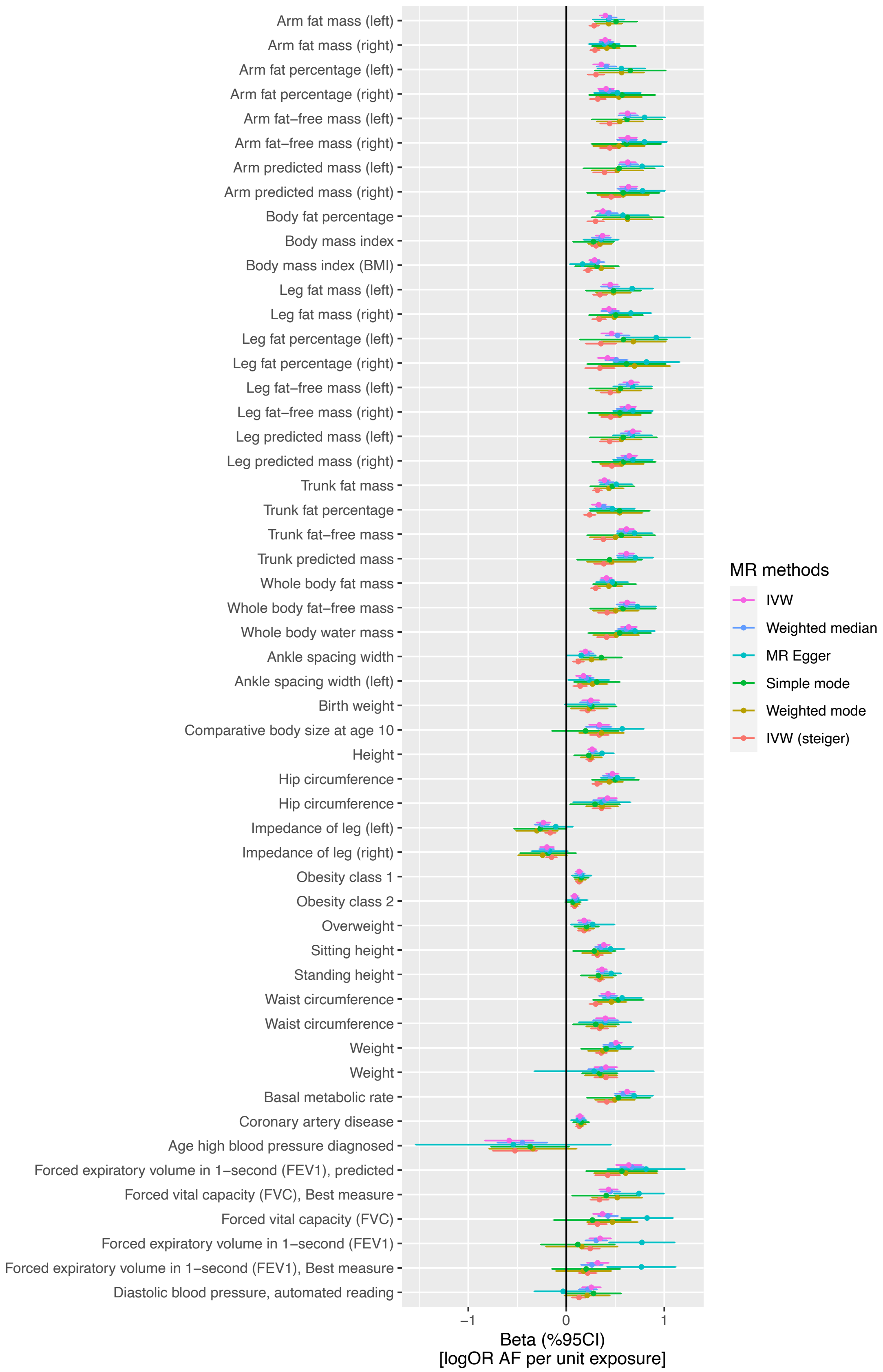

Figure S1. Sensitivity test for the significant findings in the MR analysis of phenome-wide traits to atrial fibrillation. Five different MR methods were used, including: inverse-variance weighted (IVW), MR median, MR Egger, simple mode, weighted mode and IVW with steiger filtering.

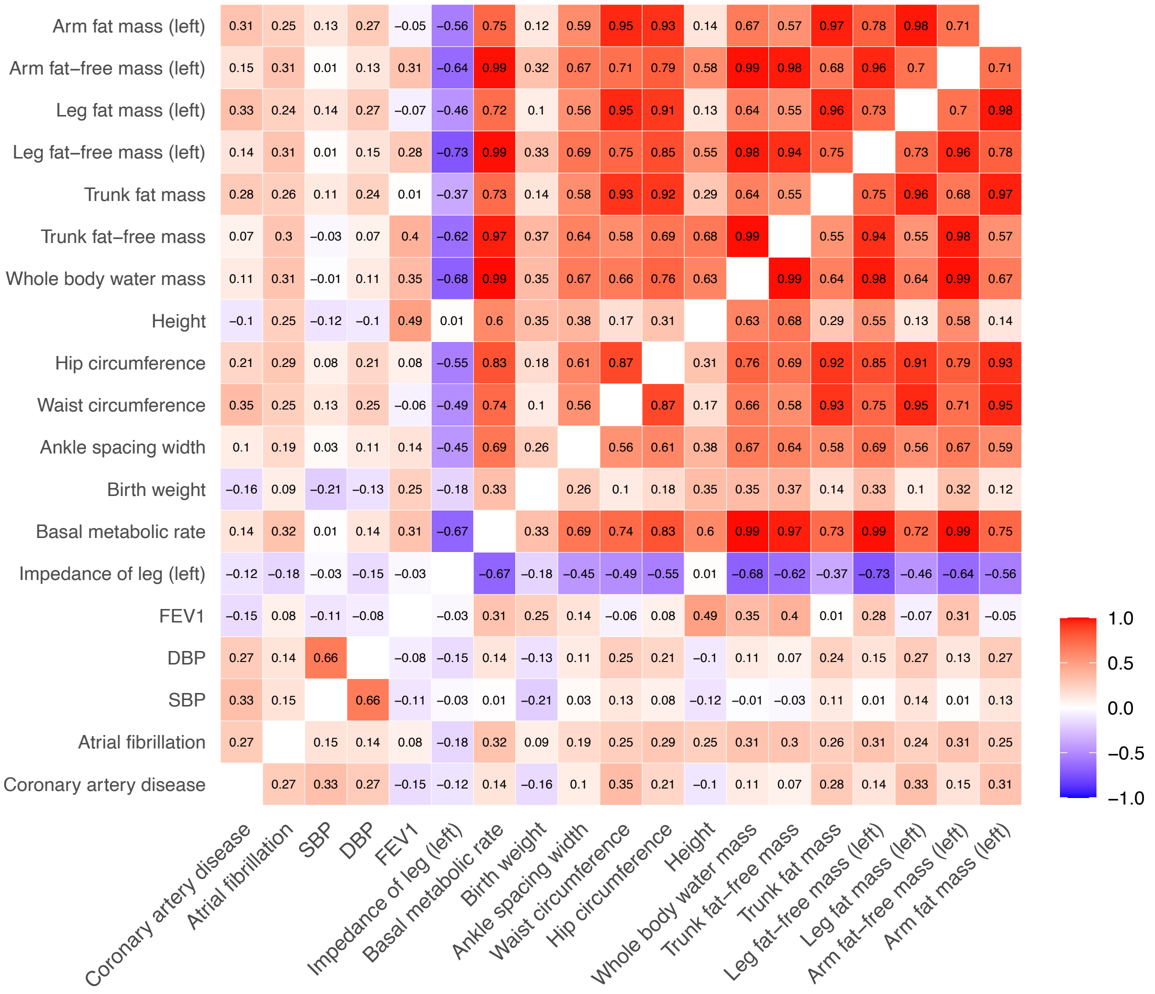

Figure S2. Genetic correlations of the risk factors. LDSC software (http://ldsc.broadinstitute.org) was used to estimate the genetic correlations and the variants were filtered to HapMap3 SNPs.

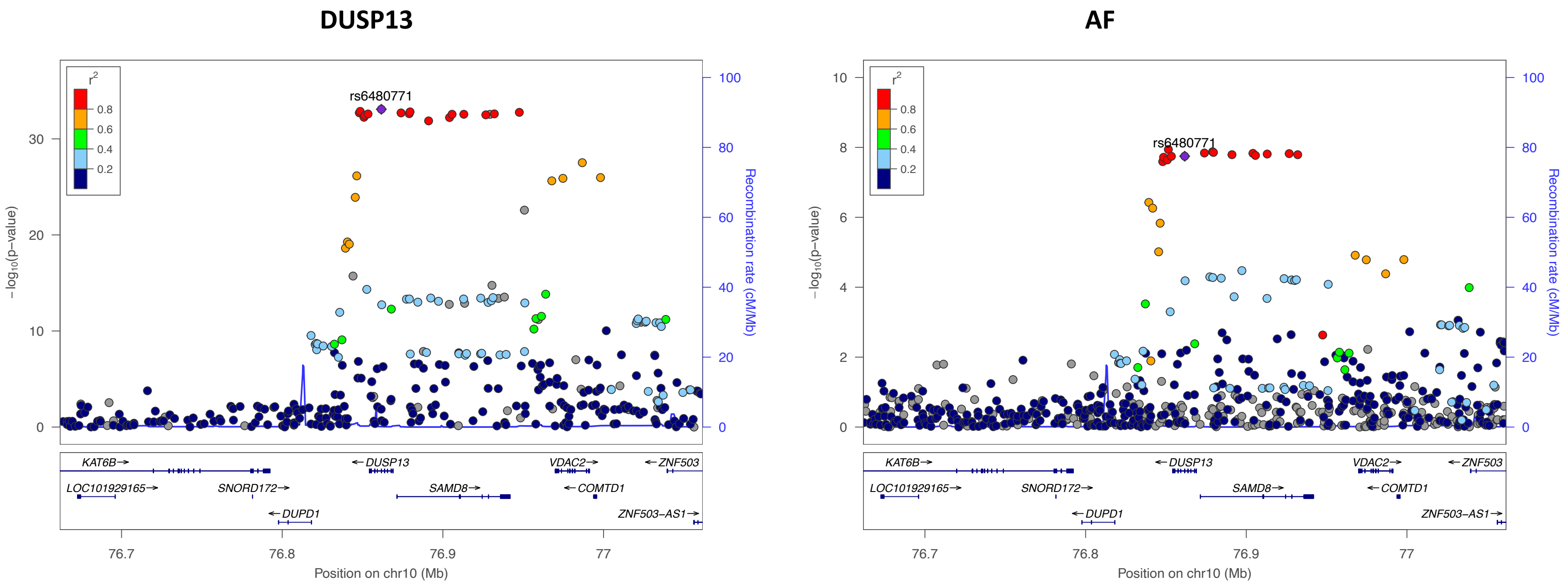

Figure S3A

Figure S3A-E. Locuszoom plots for the proteins and atrial fibrillation. For each protein, SNPs, 200kb-flanking of the *cis*-pQTL (purple diamond) at the coding gene were used. Corresponding region for AF were also shown.

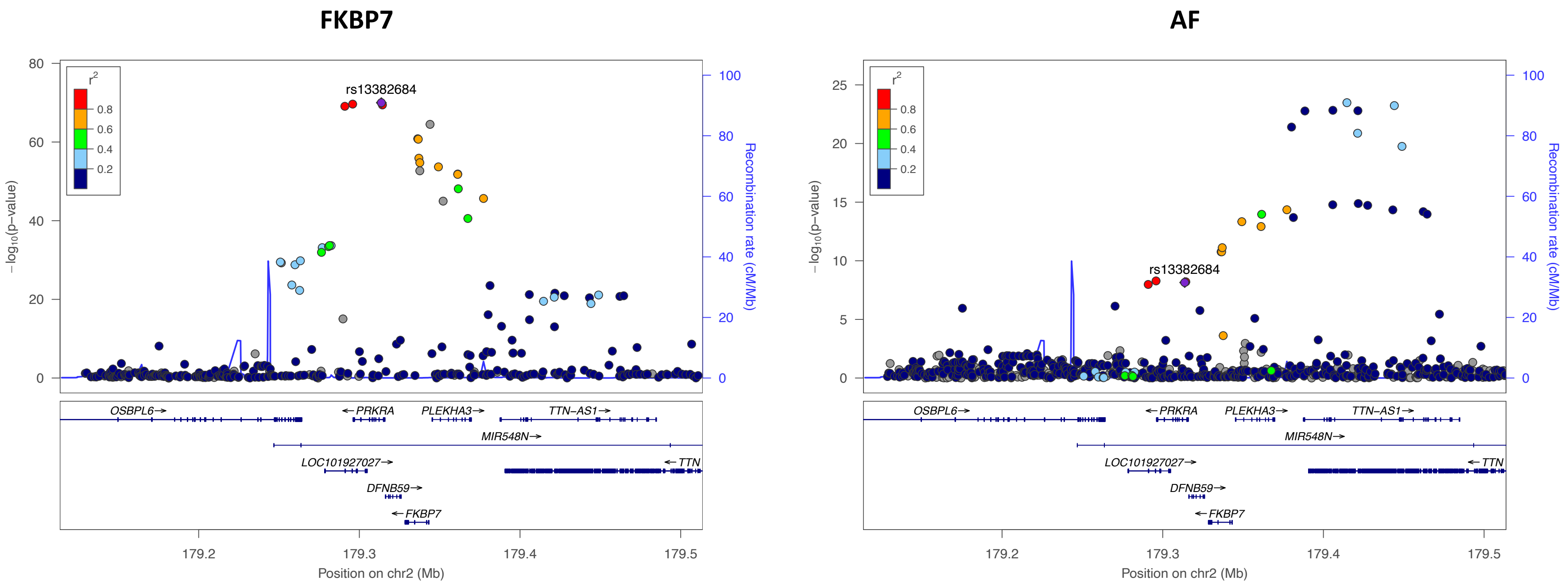
FKBP7
AF
rs13382684
r²
0.8
0.6
0.4
0.2
−log10(p−value)
Recombination rate (cM/Mb)
OSBPL6
PRKRA
PLEKHA3
TTN−AS1
MIR548N
LOC101927027
TTN
DFNB59
FKBP7
179.2
179.3
179.4
179.5
Position on chr2 (Mb)

Figure S3B

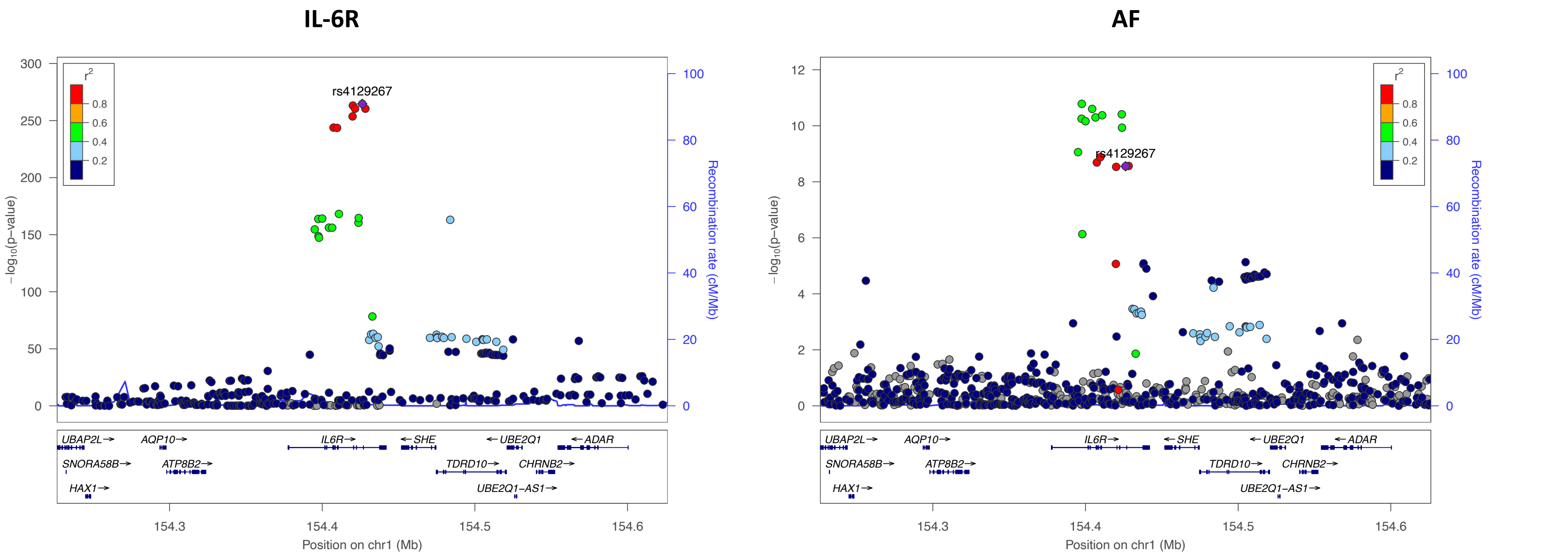
IL-6R
AF
rs4129267
r2
0.8
0.6
0.4
0.2
−log10(p−value)
Recombination rate (cM/Mb)
UBAP2L
AQP10
IL6R
SHE
UBE2Q1
ADAR
SNORA58B
ATP8B2
TDRD10
CHRNB2
HAX1
UBE2Q1−AS1
154.3
154.4
154.5
154.6
Position on chr1 (Mb)

Figure S3C

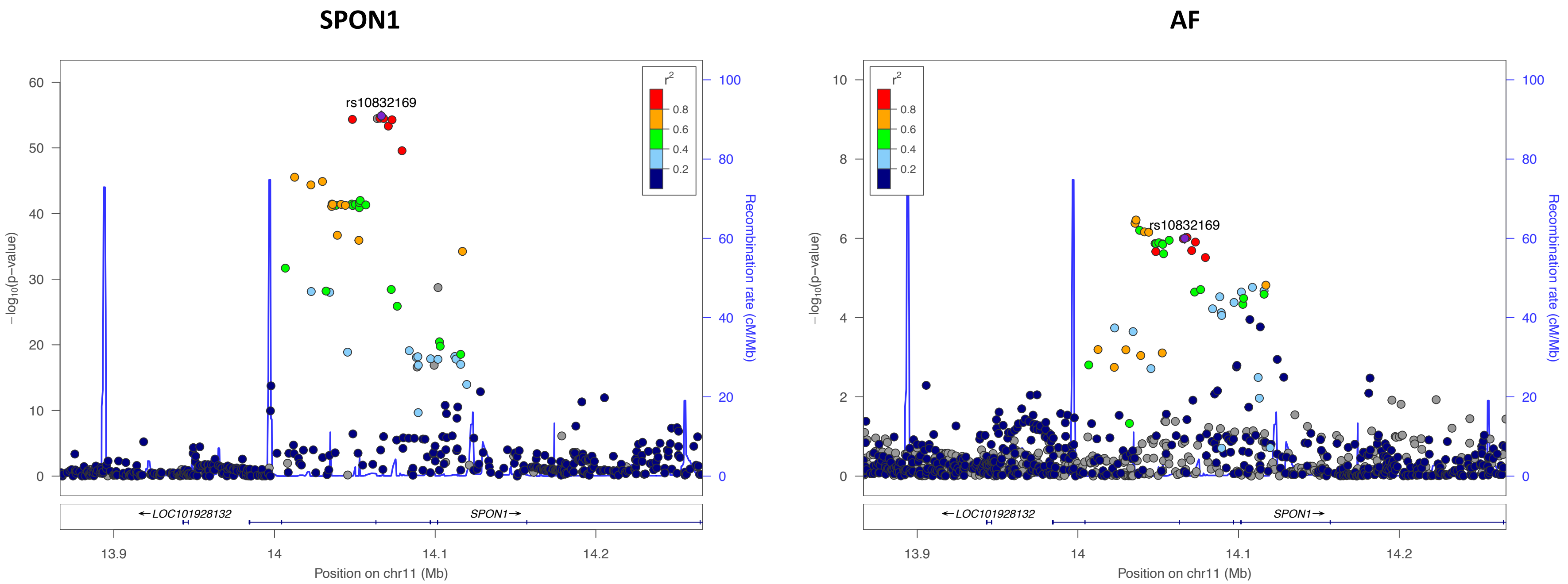
SPON1
AF
rs10832169
rs10832169
r²
0.8
0.6
0.4
0.2
−log10(p−value)
Recombination rate (cM/Mb)
←LOC101928132
SPON1→
13.9
14
14.1
14.2
Position on chr11 (Mb)

Figure S3D

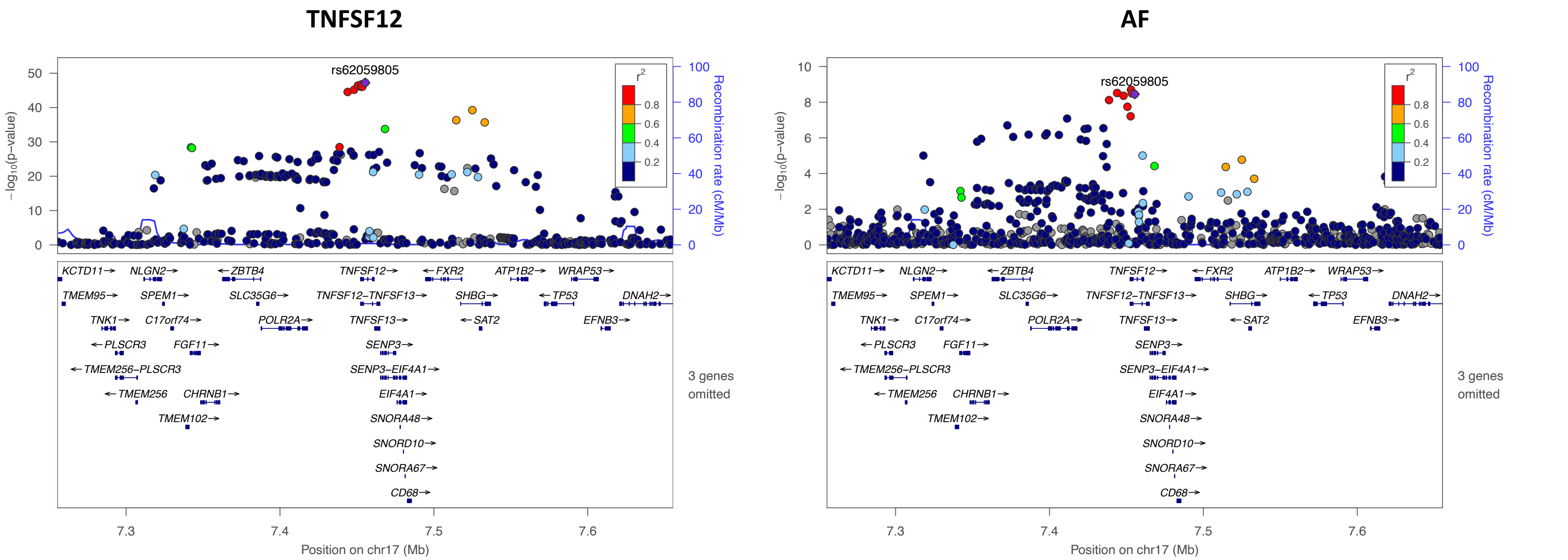
TNFSF12
AF
rs62059805
rs62059805
−log10(p−value)
Recombination rate (cM/Mb)
r2
0.8
0.6
0.4
0.2
KCTD11
NLGN2
ZBTB4
TNFSF12
FXR2
ATP1B2
WRAP53
TMEM95
SPEM1
SLC35G6
TNFSF12−TNFSF13
SHBG
TP53
DNAH2
TNK1
C17orf74
POLR2A
TNFSF13
SAT2
EFNB3
PLSCR3
FGF11
SENP3
TMEM256−PLSCR3
SENP3−EIF4A1
TMEM256
CHRNB1
EIF4A1
TMEM102
SNORA48
SNORD10
SNORA67
CD68
3 genes omitted
Position on chr17 (Mb)

Figure S3E

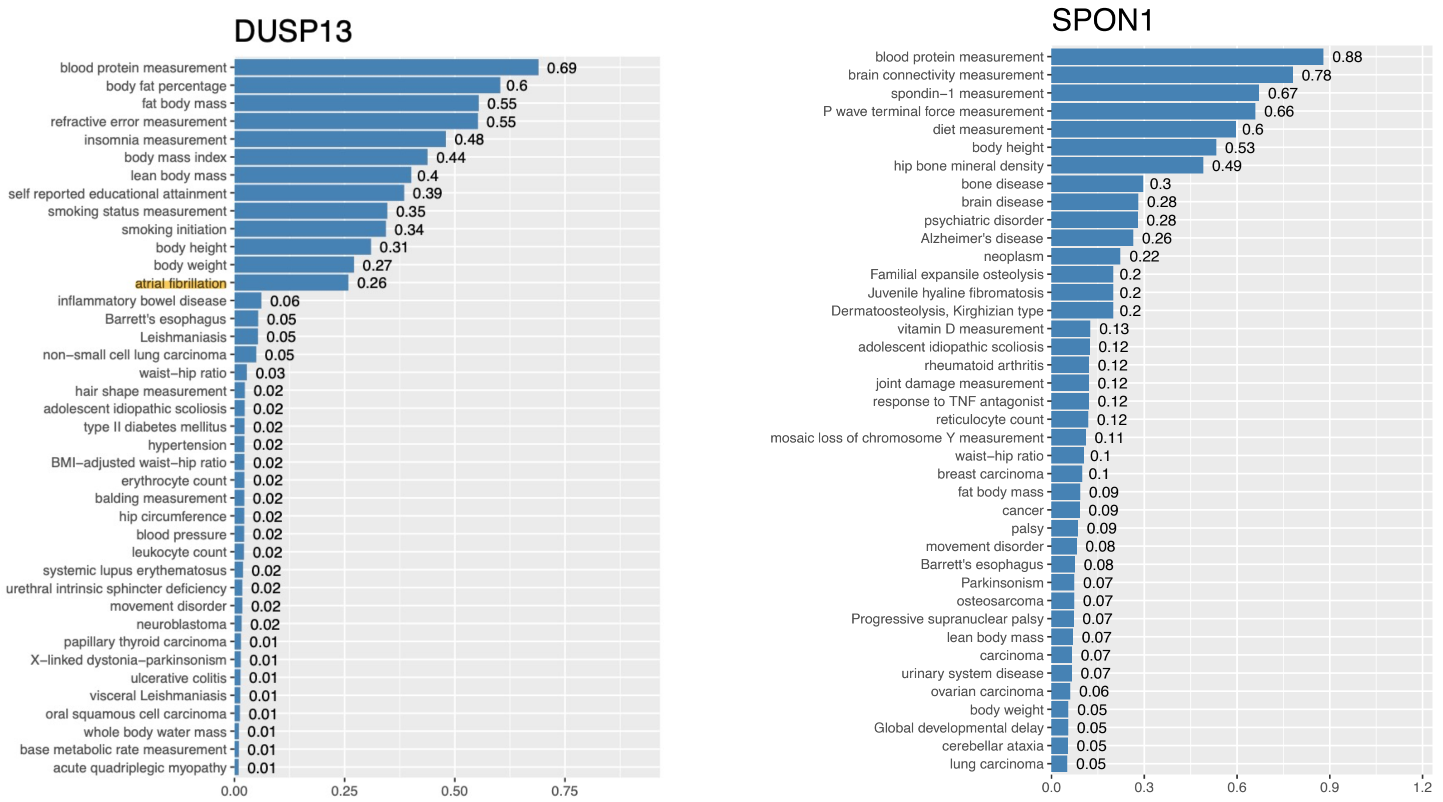

FigureS4A

Figure S4A-B. Drug targets prioritising scores. Data were obtained from Open Targets platform (https://www.opentargets.org). For each protein, the top 40 associated traits or diseases were illustrated here. The platform allows prioritisation of drug targets based on the strength of their association with a disease. It allows for the prioritisation of targets by scoring target-disease associations based on evidence from 20 data sources. Similar data sources (e.g. Open Targets Genetics Portal and PheWAS) are grouped together into data types (e.g. Genetic associations). The score for the associations ranges from 0 to 1; the stronger the evidence for an association, the stronger the association score (closer to 1). A score of 0 corresponds to no evidence supporting an association.

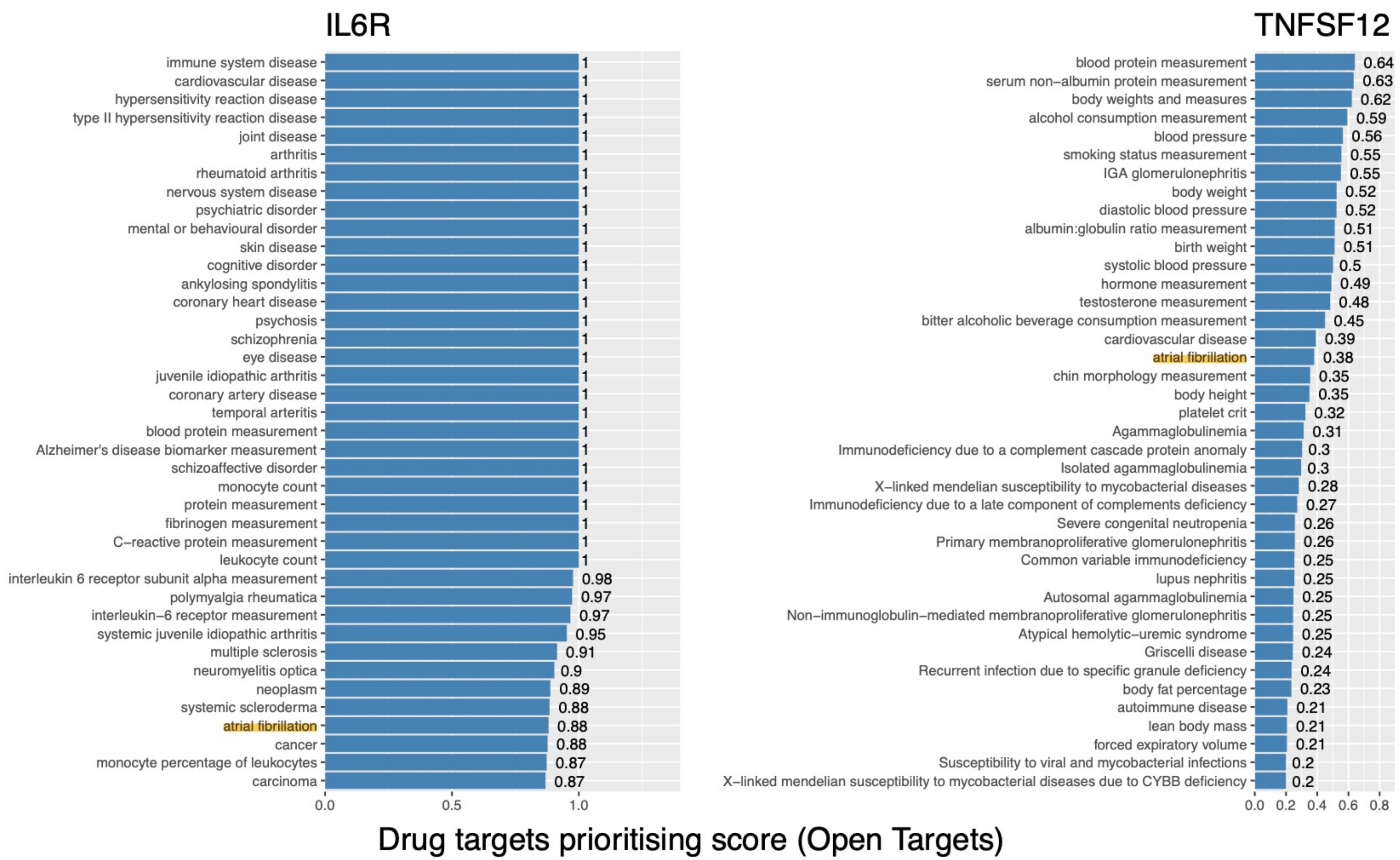
IL6R
immune system disease 1
cardiovascular disease 1
hypersensitivity reaction disease 1
type II hypersensitivity reaction disease 1
joint disease 1
arthritis 1
rheumatoid arthritis 1
nervous system disease 1
psychiatric disorder 1
mental or behavioural disorder 1
skin disease 1
cognitive disorder 1
ankylosing spondylitis 1
coronary heart disease 1
psychosis 1
schizophrenia 1
eye disease 1
juvenile idiopathic arthritis 1
coronary artery disease 1
temporal arteritis 1
blood protein measurement 1
Alzheimer's disease biomarker measurement 1
schizoaffective disorder 1
monocyte count 1
protein measurement 1
fibrinogen measurement 1
C−reactive protein measurement 1
leukocyte count 1
interleukin 6 receptor subunit alpha measurement 0.98
polymyalgia rheumatica 0.97
interleukin−6 receptor measurement 0.97
systemic juvenile idiopathic arthritis 0.95
multiple sclerosis 0.91
neuromyelitis optica 0.9
neoplasm 0.89
systemic scleroderma 0.88
atrial fibrillation 0.88
cancer 0.88
monocyte percentage of leukocytes 0.87
carcinoma 0.87
0.0 0.5 1.0
TNFSF12
blood protein measurement 0.64
serum non−albumin protein measurement 0.63
body weights and measures 0.62
alcohol consumption measurement 0.59
blood pressure 0.56
smoking status measurement 0.55
IGA glomerulonephritis 0.55
body weight 0.52
diastolic blood pressure 0.52
albumin:globulin ratio measurement 0.51
birth weight 0.51
systolic blood pressure 0.5
hormone measurement 0.49
testosterone measurement 0.48
bitter alcoholic beverage consumption measurement 0.45
cardiovascular disease 0.39
atrial fibrillation 0.38
chin morphology measurement 0.35
body height 0.35
platelet crit 0.32
Agammaglobulinemia 0.31
Immunodeficiency due to a complement cascade protein anomaly 0.3
Isolated agammaglobulinemia 0.3
X−linked mendelian susceptibility to mycobacterial diseases 0.28
Immunodeficiency due to a late component of complements deficiency 0.27
Severe congenital neutropenia 0.26
Primary membranoproliferative glomerulonephritis 0.26
Common variable immunodeficiency 0.25
lupus nephritis 0.25
Autosomal agammaglobulinemia 0.25
Non−immunoglobulin−mediated membranoproliferative glomerulonephritis 0.25
Atypical hemolytic−uremic syndrome 0.25
Griscelli disease 0.24
Recurrent infection due to specific granule deficiency 0.24
body fat percentage 0.23
autoimmune disease 0.21
lean body mass 0.21
forced expiratory volume 0.21
Susceptibility to viral and mycobacterial infections 0.2
X−linked mendelian susceptibility to mycobacterial diseases due to CYBB deficiency 0.2
0.0 0.2 0.4 0.6 0.8
Drug targets prioritising score (Open Targets)

Figure S4B

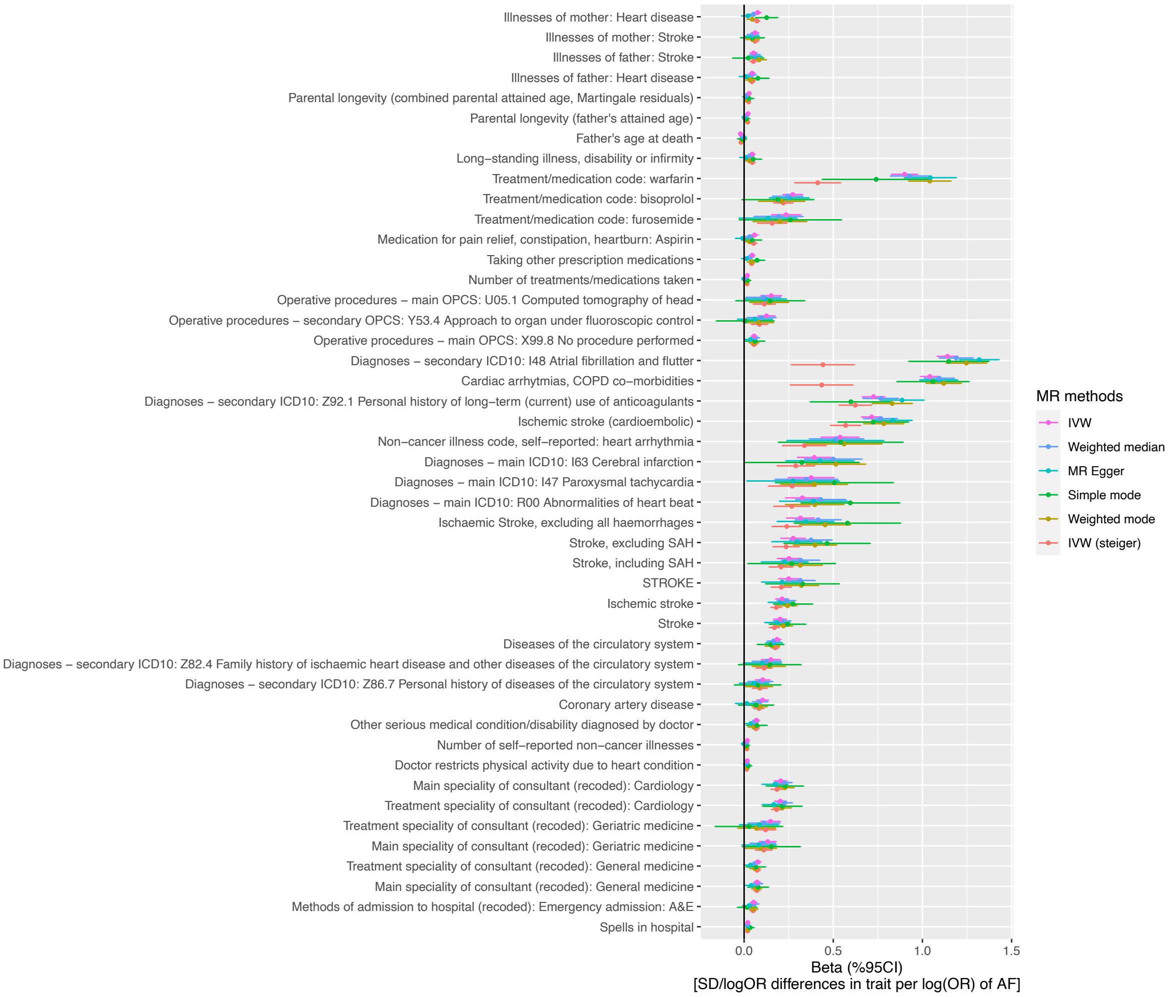

Figure S5. Sensitivity test for the significant findings in the MR analysis of atrial fibrillation to phenome-wide traits. Five different MR methods were used, including: inverse-variance weighted (IVW), MR median, MR Egger, simple mode, weighted mode and IVW with steiger filtering.
